# Targeted temperature management and cardiac arrest after the TTM-2 study

**DOI:** 10.1186/s13054-021-03718-y

**Published:** 2021-08-04

**Authors:** Fabio Silvio Taccone, Jean-Baptiste Lascarrou, Markus B. Skrifvars

**Affiliations:** 1 Department of Intensive Care, Erasme Hospital, Université Libre de Bruxelles (ULB), Route de Lennik, 808, 1070 Brussels, Belgium; 2 Service de Médecine Intensive Réanimation, Centre Hospitalier Universitaire, Nantes, France; 3 Department of Emergency Care and Services, Helsinki University Hospital and University of Helsinki, Helsinki, Finland

**Keywords:** Temperature, Cardiac arrest, Randomized trials, Outcome

## Background

The use of targeted temperature management (TTM) has been recommended for two decades in the management of patients after cardiac arrest; however, the quality of evidence behind this recommendation is moderate to low and refers only to out-of-hospital cardiac arrest (OHCA) [1–4]. Recently, Dankiewicz et al. (TTM-2 study) reported that TTM at 33 °C did not lower the incidence of death or 6-month poor neurological outcome than targeted normothermia in 1900 unconscious OHCA patients [5], with more arrhythmias resulting in hemodynamic compromise observed in the 33 °C group. There was no benefit of hypothermia in any of the prespecified sub-groups, including age, initial rhythm or duration of resuscitation. It is likely that these recent data will affect the use of TTM in clinical practice.

## TTM in cardiac arrest

How can we interpret the TTM-2 findings? The first conclusion could be the lack of benefits of TTM after cardiac arrest. Indeed, the TTM-2 study had the largest cohort of patients so far and was conducted using the best statistical methodology [5], while previous studies [1, 2] had many methodological biases (i.e., no power calculation, limited cohorts, early stopping, no blinded assessors of primary outcome, no prognostication guidelines). Also, as the general management of patients after cardiac arrest has improved over time (i.e., early recognition and treatment of the cause, hemodynamic and ventilatory management, organ support) [3], TTM might add only minimal benefits. Moreover, the experimental literature supporting the effectiveness of TTM may not be easily translated in humans, with a lack of clinically relevant long-term neurological outcomes and of reproducibility in all species [6].

A second interpretation could be that all the randomized studies on TTM after cardiac arrest are not entirely comparable (Table 1); the TTM-2 study findings would be applicable in the setting of OHCA of cardiac causes, with frequent use of bystander CPR (i.e., short no-flow time and less severe initial anoxic injury), a high proportion of patients with acute myocardial infarction and a low occurrence of shock on admission. On the contrary, in patients with an initial non-shockable rhythm due to respiratory/hypoxic causes and hemodynamic instability, the use of hypothermia at 33 °C could be considered more effective than normothermia, in particular for in-hospital cardiac arrest [4]. Secondary analyses of the TTM-2 study and individual patient meta-analyses from available datasets of randomized trials may identify some subgroups of patients who are better candidates for TTM and should be considered for future research.

**Table 1:** Characteristics of the clinical randomized clinical studies comparing hypothermia at 33 °C with standard of care (i.e., no temperature control or normothermia) or 36 °C targets. For continuous variables, data are presented as mean $\pm$ SD or median (IQRs), as available in the main manuscript

	Dankiewicz et al. [5]	HACA group [2]	Bernard et al. [1]	Nielsen et al. [7]	Lascarrou et al. [4]
Design	Multicentric	Multicentric	Single-Centre	Multicentric	Multicentric
N (HT group)	1861 (930)	275 (138)*	79 (43)**	939 (473)	584 (284)
Age, years	64 ± 13	59 (49–67)	67 (49–89)	64 ± 12	67 (57–76)
Male gender	80%	77%	58%	83%	65%
OHCA	100%	100%	100%	100%	74%
Bystander CPR	82%	49%	49%	73%	70%
Shockable rhythm	72%	96%	100%	79%	0%
Time to ROSC, min	25 (16–40)	22 (17–33)*	27 ± 13	25 (18–40)	18 (10–25)
Cause of Arrest	Cardiac/UNK	Cardiac	Cardiac	Cardiac/UNK	All**
Shock on Admission	28%	49*	NR	15%	56%
STEMI on Admission	41%	NR	NR	40%	16%
Lactate, mmol/L	5.9 ± 4.4	NR	8.3 (2.2–14.9)	6.7 ± 4.5	5.8 (3.2–9.0)
Outcome Assessment	6 months	6 months	Hospital Discharge	6 months	3 months
Mortality, %*	50%	41%	51%	50%	81%
UO Assessment Scale	mRS 4–6	CPC 3–5	CPC 3–5	CPC 3–5	CPC 3–5
UO, %	55	45	51	54	90
Prognostication Rules	Present	Absent	Absent	Present	Present
Generalisability/Bias	High/low	Low/high	Low/high	High/low	High/moderate

HACA, Hypothermia After Cardiac Arrest; N, total number of included patients; HT, hypothermia; OHCA, out-of-hospital cardiac arrest; CPR, cardiopulmonary resuscitation; ROSC, return of spontaneous circulation; min, minutes, UNK, unknown; STEMI; ST-elevation myocardial infarction; UO, unfavorable neurological outcome; mRS, modified Rankin scale; CPC, Cerebral Performance Category

*In the hypothermia group

**Mainly respiratory causes

A third interpretation could be the limited applicability in common practice. The TTM-2 study had high patients’ heterogeneity (i.e., shockable and non-shockable rhythms; no age limit), a very short no-flow time and a large number of bystander-initiated resuscitation (implying a limited brain injury), which are not common characteristics of CA patients in many registries. Nonetheless, the TTM-2 study included patients in a higher number of countries and several continents when compared to other studies [1, 4] (i.e., high generalisability); also, time to resuscitation, time to target temperature and rewarming rate were comparable to previous trials [2, 4] (i.e., TTM was provided similarly than in studies reporting benefits from hypothermia); the less strict inclusion criteria (i.e., including some unwitnessed cardiac arrest or non-shockable rhythm as well as elderly patients, i.e., > 75 years of age) than other studies [2, 4] allowed a larger proportion of eligible patient to be enrolled (i.e., reduced selection bias); the presence of strict prognostication rules minimized the risks of limitations of life-sustaining therapies decided by unblinded investigators [1, 2] (i.e., reduced performance bias).

A fourth interpretation could be the large inclusion of patients with extreme patterns of brain injury, in whom TTM would provide only minimal benefits. The TTM-2 study required randomization within 3 h from return of spontaneous circulation (ROSC) to avoid late application of hypothermia, so that patients with mild brain damage were also included. Also, patients with irreversible brain injury and no chance of recovery regardless of different therapeutic interventions might also be enrolled. Future studies are needed to identify prognostic tools able to quantify earlier the severity of hypoxic brain injury and to identify those patients in whom neuroprotective strategies, including TTM, should be evaluated.

How should we use then TTM after cardiac arrest ? Clinicians should consider that the “normothermic” arm (i.e., aiming for a temperature below 37.8 °C) in the TTM-2 study required use of sedation for 40 h in all patients and active temperature management (i.e., cooling devices with temperature-feedback control; relatively slow rewarming and avoidance of fever for 72 h) in almost 50% of them. As such, abandoning any temperature control protocol in cardiac arrest patients is not acceptable. After the publication of the TTM study in 2013 [7], observational studies have already indicated that changes temperature targets to 36 °C were associated with a less accurate TTM delivery and worse outcomes when compared to 33 °C target [8]. Temperature control after CA remains a complex and time-consuming intervention, which, if inadequately applied (i.e., no sedation, no active temperature and fever control) might result in unpredictable temperature trajectories and in a less precise patients’ management, with deleterious effects on neurological outcome. Interestingly, none of these randomized studies could provide a “high quality” TTM [9], because of human and technical constraints which could alter speed of cooling, reduce the accuracy of temperature control, shorten the rewarming period or expose patients to prolonged post-TTM fever. Although high-quality TTM was not an independent predictor of favorable neurological outcome in a recent study [10], additional research is required to understand how high-quality TTM could influence the results observed in large randomized trials.

## Conclusions

Physicians treating patients resuscitated after OHCA should consider that active maintenance of normothermia (i.e., specific protocol for sedation, prognostication and active temperature control) appears to be the optimal strategy for cardiac arrest of cardiac origin and with early resuscitation. Whether TTM at 33 °C still could be indicated in non-shockable rhythms due to non-cardiac causes remains to be considered. Future studies in in-hospital cardiac arrest are warranted. Abandoning TTM completely is not an option.

## Data Availability

Not applicable.

## Abbreviations

CPR: Cardiopulmonary resuscitation
ICU: Intensive Care Unit
OHCA: Out-of-hospital cardiac arrest
ROSC: Return of spontaneous circulation
TTM: Targeted temperature management
